# Acceptability of the power over pain portal among patients awaiting tertiary care consultation: A qualitative study of patients’ perceptions

**DOI:** 10.1177/20552076241288748

**Published:** 2024-10-07

**Authors:** Alesha C King, Amin Zahrai, Etienne Bisson, Yaadwinder Shergill, Pavel Andreev, Rachael Bosma, Alexandra O MacNeil, Arun Radhakrishnan, Joshua A Rash, Rosemary Wilson, Patricia Poulin

**Affiliations:** 1 Department of Psychology, 7512 Memorial University of Newfoundland, St John's, Newfoundland, Canada; 2 10055 Ottawa Hospital Research Institute, Ottawa, Ontario, Canada; 3 School of Epidemiology and Public Health, 6363 University of Ottawa, Ottawa, Ontario, Canada; 4 71459 Kingston Health Sciences Centre, Kingston, Ontario, Canada; 5 School of Rehabilitation Therapy, 4257 Queen's University, Kingston, Ontario, Canada; 6 Telfer School of Management, 6363 University of Ottawa, Ottawa, Ontario, Canada; 7 Centre for the Study of Pain, 7938 University of Toronto, Toronto, Ontario, Canada; 8 7985 Women's College Hospital, Toronto, Ontario, Canada; 9 12361 Faculty of Medicine, Dalhousie University, Halifax, Nova Scotia, Canada; 10 Department of Family Medicine, 6363 University of Ottawa, Ottawa, Ontario, Canada; 11 Department of Family and Community Medicine, 7938 University of Toronto, Toronto, Ontario, Canada; 12 School of Nursing, 4257 Queen's University, Kingston, Ontario, Canada; 13 Department of Anesthesiology and Pain Medicine, 6363 University of Ottawa, Ottawa, Ontario, Canada; 14 The Ottawa Hospital Pain Clinic, Ottawa, Ontario, Canada

**Keywords:** Chronic pain, self-management, telemedicine

## Abstract

**Objective:**

Chronic pain affects approximately 7.6 million Canadians and access to care remains an issue. The Power Over Pain (POP) Portal offers immediate access to evidence-based resources ranging from low- (e.g. education, self-management), to high- (e.g. individual counseling) intensity. We explored the POP Portal's acceptability, usability, and perceived usefulness among patients newly referred to a tertiary care pain clinic.

**Methods:**

We used a descriptive, qualitative approach with a prospective cohort of 60 adult patients recently referred to The Ottawa Hospital Pain Clinic. Patients were offered an orientation session and asked to participate in a seven-week follow-up interview. Data were thematically analyzed in an iterative process, whereby responses were reviewed and coded by two members of the research team.

**Results:**

Of the 60 patients referred to the POP Portal by clinic clerks, 45 participated in the orientation session, and 40 completed a four-week follow-up. All 40 patients had used the POP Portal and recommended that we continue to offer the POP Portal to patients awaiting care. We identified overarching themes of acceptability (five subthemes), usability (ten subthemes), accessibility (three subthemes), and patient value of the POP Portal (three subthemes). This includes (1) the POP Portal provides easy access to chronic pain resources; (2) the POP Portal is helpful in developing an understanding of chronic pain; and (3) improvements to the POP Portal are needed to increase usability and foster a user-friendly experience.

**Conclusions:**

The POP Portal offers accessible and diverse resources for people living with pain awaiting a tertiary care consultation; however, patients would like to see resources specific to diagnosis. Improvements are suggested to allow greater increase the POP Portal usability.

Chronic pain (CP) affects approximately 7.6 million youth and adults living in Canada.^1^ Access to care can be challenging; more than 50% of patients referred to specialized programs wait more than 6 months (the threshold for medically unacceptable wait times),^2^ with some patients waiting years or never being seen because of clinic inclusion/exclusion criteria.^3^ Digital self-management resources have robust evidence attesting to their effectiveness in reducing pain severity and improving quality of life among people living with pain (PLWP)^4^; however, these resources are not localized, and can be difficult to find.

The Power Over Pain (POP) Portal (poweroverpain.ca) is a digital platform for PLWP, codesigned by a team of researchers, healthcare providers, and PLWP. The Portal was created by and for Canadians, but it is freely accessible worldwide to anyone with an internet access, providing access to symptom self-assessments, personalized feedback on outcomes, and access to a collection of free evidence-informed interventions. These interventions include low-intensity resources such as education (e.g. LivePlanBe+ and PainU Online), medium-intensity resources including internet-delivered pain self-management courses, based on acceptance-commitment therapy or cognitive behavioral therapy (e.g. the Pain Course^5–9^), peer support, and live or recorded virtual workshops, and high-intensity resources such as individual counseling through Wellness Together Canada.^10^ Together, these interventions can be curated for the management of a host of concerns (e.g. sleep, mood, movement). Resources are selected for the POP Portal by Lived Experience and Scientific Advisory Committees based on existing evidence of the resource, delivery, location (i.e. virtual, province specific, Canada specific), and topic (e.g. sleep quality, diagnosis, medications and substance use, financial aid). All resources are required to be free of cost to be included on the platform. The POP Portal's Lived Experience Advisory Committee consists of PLWP who meet weekly to discuss the development and direction of the POP Portal, providing insight to the Scientific Advisory Committee on how the Portal may be improved to meet the needs of PLWP. The Scientific Advisory Committee, consisting of researchers and healthcare professionals, takes this feedback to iteratively refine the POP Portal using an evidence-informed approach. Together, the two committees collaborate to have a digital platform which is curated for PLWP by PLWP while maintaining a stringent level of quality.

The Ottawa Hospital (TOH) Pain Clinic recently adapted and implemented the Stepped Care 2.0 framework^11^ which resulted in improved access to a range of care options for patients.^12^ Wait-times to access the pain clinic orientation session, which then provides access to several of the pain clinic's programs is between 2 and 3 months; but patients may wait several more months to access some of the clinic services. There remain windows of opportunity to support patient engagement with different treatments and resources to improve their overall ability to cope with pain and improve their well-being. As such, TOH Pain Clinic leadership is interested in integrating the POP Portal into patient's care pathway as part of ongoing quality improvement initiatives. The National Implementation Research Network (NIRN) offers guidance on the sequential steps to implementing programs into clinical settings, including (1) exploration of the problem and potential evidence-based solutions; (2) adapting the system for program installation; (3) initial implementation and making changes to the practice; and (4) full implementation of the program into the system as standard practice.^13^ Aligning with NIRN's third phase of implementation,^13^ the present study documents a pilot implementation of the POP Portal into the referral pathway of TOH Pain Clinic and evaluates the POP Portal acceptability and usability among patients using the Portal while awaiting their first visit.

## Methods

### Study design and setting

The present study is a qualitative observational implementation study, for the implementation of the POP Portal at TOH Pain Clinic, a tertiary care pain clinic in Ottawa, Ontario, Canada. This study received research ethics board exemption through a quality improvement stream by the Ottawa Health Science Network Research Ethics Board. We used the Standards for Reporting Qualitative Research and Consolidated Criteria for Reporting Qualitative Research reporting guidelines when writing our report.^14,15^

### Patients

Patients were invited to participate in this study by TOH Pain Clinic clerks after receiving a referral to the clinic (and prior to triage process). Patients referred from January 2023 to March 2023 were screened for eligibility by clinic clerks and included (1) being 18 years of age or older; (2) had CP (ongoing, persistent, or recurrent pain for more than three months^16^); (3) spoke French or English; (4) had access to the internet via computer, phone, or tablet; and (5) agreed to participate in the study. Eligible patients, up to a total of 60, were invited to participate in the study, whereby clinic clerks informed the patient there was an online platform called the POP Portal which offers free online pain self-management resources. Clinic clerks further asked if the patient was interested in an orientation session; if so, their information was forwarded to the research assistant. Given the aim of the study, the specificity of questions asked, application of theory, brevity of dialogue, and cross-case analysis, we anticipate reaching saturation and good information power within 60 participants.^17^

### Procedure

**Figure 1.**
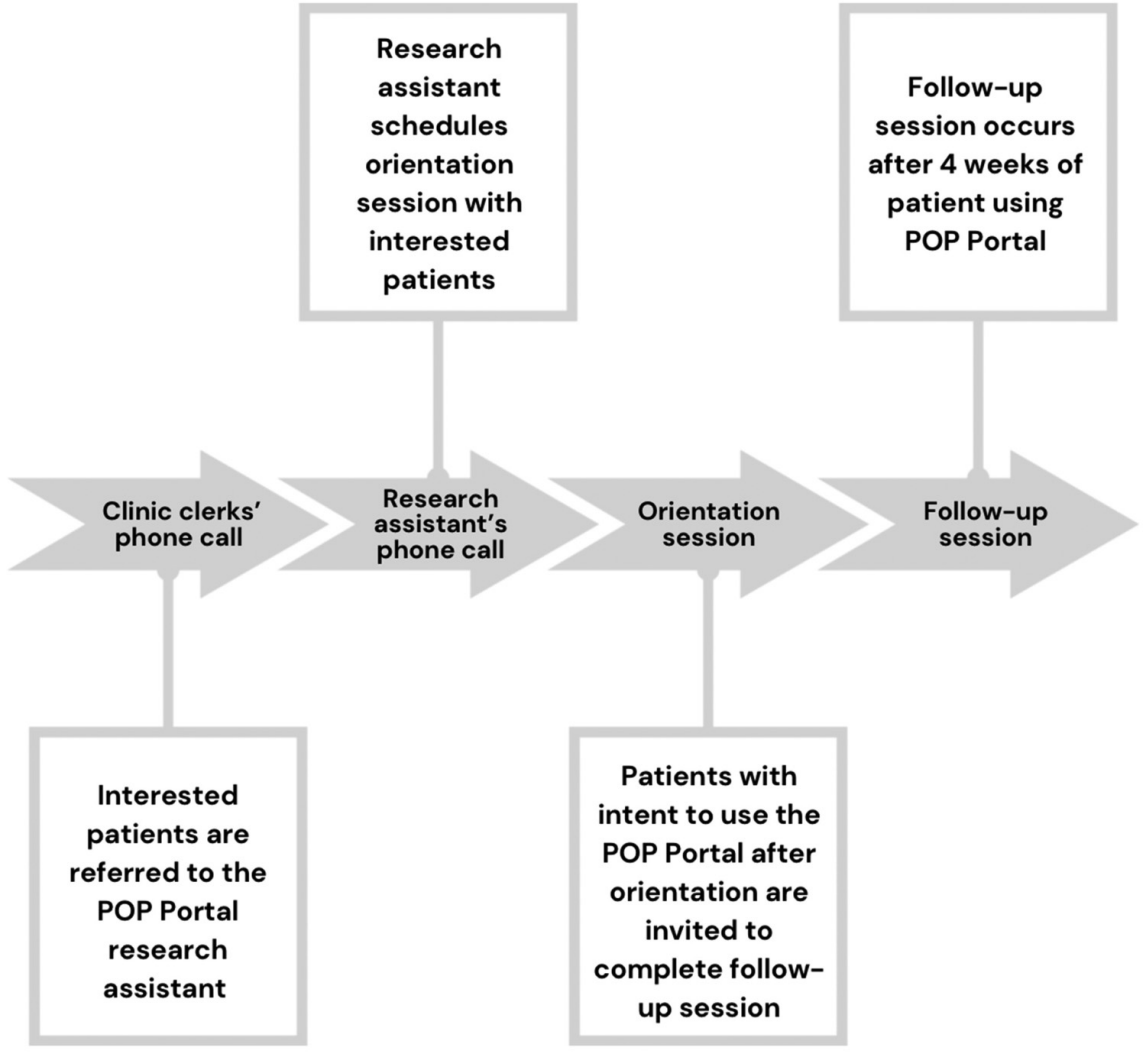
Summary of procedures.

Patients who expressed interest in the POP Portal to the TOH Pain Clinic clerk were contacted via phone by a research assistant (AZ). The research assistant introduced themselves as a research assistant from TOH and confirmed the patients’ interest in being contacted about the pilot project. Information was provided about the pilot project and offered a POP Portal orientation session (refer to Figure 1). Patients provided verbal informed consent prior to participating in the study, an approved form of consent by TOH Research Institute. The orientation session was conducted by the research assistant, spanned 10–15 min, and involved an introduction to the POP Portal, navigation, and account creation as an optional enhancement feature. Patients were asked if they intended to use the Portal and informed about the opportunity to participate in a follow-up interview. Patients who attended the orientation session and agreed to a follow-up session used the POP Portal with no set instructions. Patients were contacted by the research assistant to complete an interview after a four-week period.

### Measures

Demographic information (e.g. age, sex, gender, date of referral to the pain clinic, and pain diagnosis) was collected from patient's medical records. Information pertaining to patient retention throughout the study was collected during the orientation session and at follow-up. This included: (1) interest in learning about POP; (2) completion of the orientation session; (3) intent to use POP (post-orientation); and (4) actual Portal use (at follow-up session).

Semistructured interviews were conducted with patients who agreed to take part in the follow-up session to evaluate the acceptability and usability of the Portal (refer to Appendix A for interview guide). The interview used a grounded theory approach, targeting patient experience, intent to use the POP Portal, and areas of which the POP Portal may be improved. 1:1 interviews were conducted by a male research assistant (AZ) using the videoconferencing software MS Teams and lasted up to 15 min. Field notes were taken by the research assistant to document patient responses to questions for later analysis. Research assistants within this study are trained in interviewing methods and are supervised by a registered psychologist (PP).

### Analysis

Descriptive statistics were conducted using SPSS Statistics software to describe sample characteristics and patient retention throughout the study. Characteristics of patients who did or did not participate in an orientation session were evaluated using independent samples *t*-test for age and chi-square test of independence for sex and back pain/symptoms of radiculopathy. Interviews were reviewed by two members of the research team (AZ and EB) who then compared notes and organized responses as positive, constructive, negative, or neutral to aid interpretation. Interviews were then coded and thematically analyzed by the research team (AZ, EB, AK), developing overarching themes across emerging codes. Codes were categorized into their respective themes based on their alignment with acceptability (informed by the Theoretical Framework of Acceptability,^18^ illustrating seven components which come together to determine the acceptability of an intervention, including affective attitude, burden, ethicality, intervention coherence, opportunity costs, perceived effectiveness, and self-efficacy), accessibility and usability (informed by the System Usability Scale,^19^ providing insight on the effectiveness, efficiency, and satisfaction of using the intervention), and perceived value of the POP Portal among PLWP.^20^ Disagreements on coding of specific items resulted in a meeting between members of the research team until an agreement was made.

## Results

### Sample characteristics

Descriptive statistics are presented in Table 1. A total of 60 patients were referred to the POP Portal research assistant by the clinic clerk. The majority of referred patients were female (*N *= 26, 63.4%), self-identified as woman (*N *= 26, 63.4%), and were diagnosed with neuropathic pain (*N *= 31, 75.6%) and/or musculoskeletal pain (*N *= 24, 58.5%).

**Table 1.** Sample characteristics.

	Completed follow-up	
Characteristic	*N*	Proportion (%)
Sex		
Male	14	34.1
Female	26	63.4
Gender		
Man	14	34.1
Woman	26	63.4
Pain Diagnosis		
Musculoskeletal	24	58.5
Visceral	3	7.3
Neuropathic	31	75.6
Post-traumatic/ surgical	3	7.3
Other	1	2.4
	*M*	*SD*
Age	54.62	15.74

*Note*. Patients may be diagnosed with multiple types of pain and is reflected in the study's demographics. Characteristics of patients who completed follow-up are representative of the total sample.

**Figure 2.**
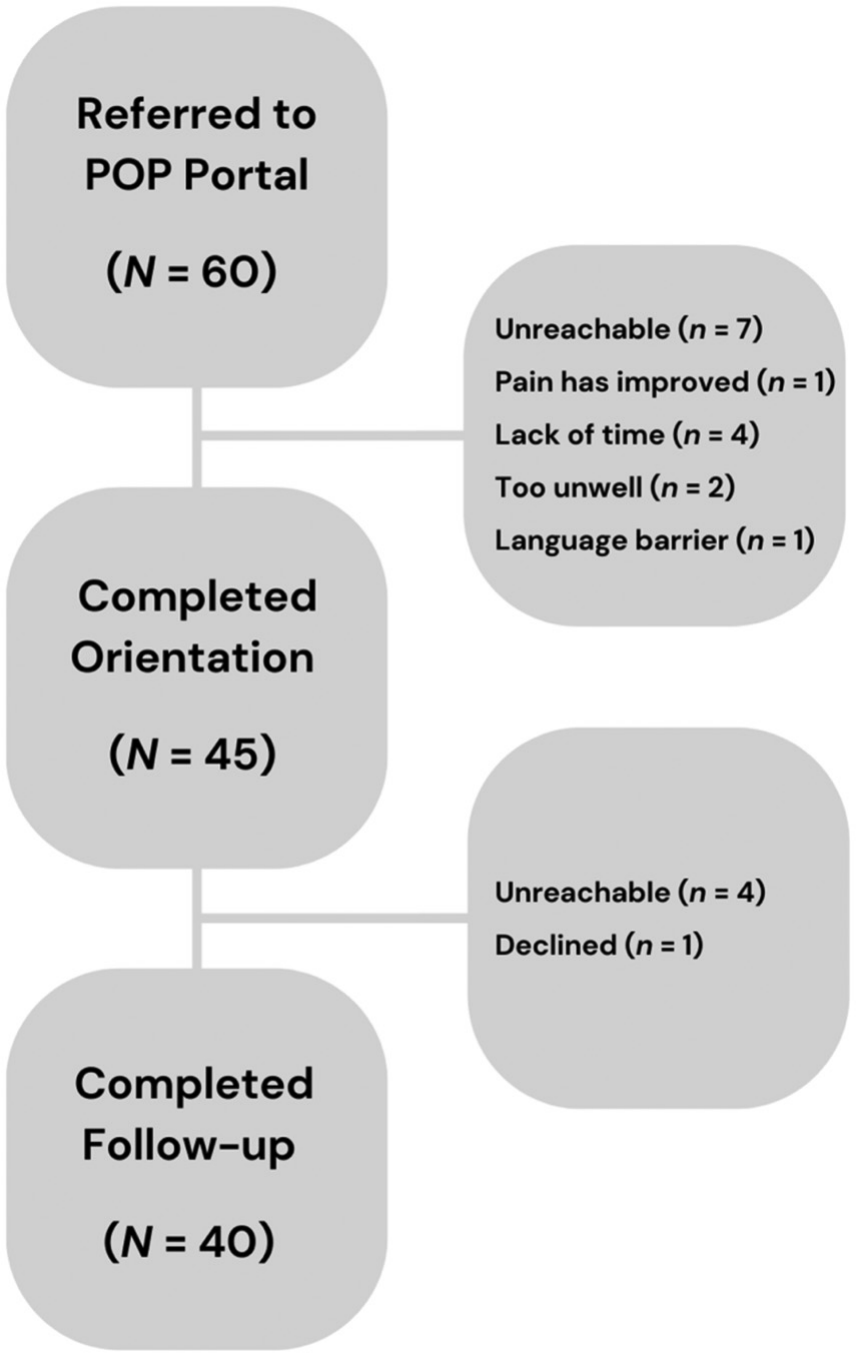
Flow diagram of patient retention.

Out of the 60 patients referred to the POP Portal research assistant, majority (*N *= 45; 75%) attended an orientation session and indicated their intention to adopt the Portal to support their pain management, seven were unreachable, one indicated improvement in pain symptoms and did not wish to participate, four patients indicated lack of time, two patients were too unwell, and one experienced a language barrier impacting ability to participate. There were no statistically significant associations between participation in the POP Portal orientation sessions and age (*p *= 0.11), sex (*p *= 1.00), or presence of back pain and/or symptoms of radiculopathy (*p *= 0.22) of participants. Forty of the 45 patients participated in the follow-up interview, refer to Figure 2. Patients were asked whether they had used the POP Portal over the past four weeks, which all 40 participants had. Further, when asked if they planned to continue to use the POP Portal in their CP management regime, 80% of participants (*N *= 32) said yes.

### Semistructured interviews

A summary of the qualitative findings is presented in Table 2. Saturation was reached after the completion of 40 interviews, whereby no new information was presented.

**Table 2.** Summary of findings.

Subtheme	Definition	Number of times endorsed	Quotes
Accessibility			
Offers accessible care	Patients report easy access to chronic pain management resources at the time of need.	8	*Some people have no other resources/sources to go to and the Portal can be good for them.*
			*There's no other website that offers so many resources all in one place; I liked this about this platform.*
			*I am living by my own and need to otherwise find resources for my pain management.*
			*Homewood Health was something tangible that I can use as an immediate intervention.*
			*Amalgamation of resources and vast amount of information and free resources on the Portal are good for mental health needs.*
			*A face-to-face appointment at the pain clinic would be better but the Portal is so much better than doing research on your own and trying to find your own resources. When you are in pain, your motivation for those sorts of things become greatly reduced.*
			*Life get's busy and reaching out to find self-management tools for chronic pain on the web takes time. I am glad the Portal is being offered to PLWP to help them out.*
			*The Portal is a resource that you can access from home/work/anywhere.*
Offers alternative treatments	The POP Portal offers chronic pain management that does not involve pharmaceuticals.	1	*Much information provided to PLWP to help them without the use of drugs.*
Need for additional resources	Patients indicate they would like to see additional resources on the POP Portal, including substance use, surgery information, and resources on time management.	2	*A suggestion for improvement would be virtual access to a pain specialist, but I realize there are complications in that being a reality. I think the portal is useful, in terms of providing information, however most people are going to want some guidance from a human being.*
			*I think having resources on time management on the Portal can be useful (something to improve on).*
Usability			
Content is engaging	Patients remained engaged with the POP Portal during use.	1	*Podcasts from healthcare professionals and PLWP as well as neuroscience education were interesting.*
Improvements should be made for visual accessibility	Some aspects of the website may be difficult to read due to font size.	1	*Could we have a button to change the size of letters on the Portal pages?*
Fluidity across sites should be improved	Some patients report issues moving between external pages and the POP Portal.	2	*Users can get in and get information from resources on the Portal but need to make new credentials for some resources (e.g. LivePlanB+). Is there a way to export your credentials from one website to the next.*
			*For resources that are hosted by external organization (e.g. TAPMI), is there way to make the Portal be connected to those pages so that it allows users to go back to the Portal without losing the Portal's page? Something like a back button or a pop-up message that says: “To go back to the PoP Portal, click here”.*
Additional site engagement tools	Patients would like to see more interactive and engaging content within the POP Portal. This may include animations, printed materials, and visuals.	3	*I suggest including more animated content and having more blocks instead of empty space in the Portal.*
			*So far what I need is in the Portal and it's not missing anything. I think optional evaluations to test the patient's knowledge might be nice to have at the end of a module or course but need to be careful not to add stress for patients either.*
			*Interested in printed materials of the Portal when we produce these.*
Improvements should be made to organization of resources to increase usability	Patients would like to see better organization of resources, and options to save favored resources for a later time.	3	*make the resource cards appear while you are logged in; have a scrolling bar to show all resources that are in one category; have a way to search content on the Portal like a search bar*
			*If your Portal offered something like this [a diagnosis-specific filter] for users when they start using it, it would be useful: I have a sore back and these are the causes and the Portal directs you to these areas.*
			*Can we put in a way for Portal users to bookmark/tag their favorite resources after they go through them?*
Site glitches and computer issues	Some patients report technical issues concerning the POP Portal, including missing logos, connection issues, and missing information.	2	*The Portal's logo is missing from the website favicon (image displayed on left side of tabs at the top of a web browser).*
			*I had issues using my computer and connecting to the Portal.*
POP Portal was too structured	The POP Portal appeared too formal and structured to the patient.	1	*I find the Portal to be a bit too formal and too structured. It has a lot of empty space between resource cards.*
Time constraints which impede ability to engage with POP Portal	Patients report not having sufficient time in their daily schedules to effectively engage with the POP Portal.	3	*I am not feeling well due to my severe medical condition and have many things going on with lawyers; won't have time to complete the Portal on time and doesn't like to have an appointment scheduled because I feel it pressures me to comply and complete tasks in timeframe that wouldn't work for me.*
			*I have little free time with my new job and business; used the Portal but won't have extra time for the Portal at this time.*
			*As a healthcare professional, I haven’t used the Portal much yet due to busy clinic schedules. I have promoted the Portal to my healthcare provider colleagues though.*
POP Portal was too complex	The POP Portal provides a variety of information that may become complex and overwhelming for some patients.	1	*The Portal is good in theory but not in practice; for example, it's good for teaching doctors how to deal with patients with a specific type of pain. But for patients, it's either too much info or it's not as much empathy involved. Not enough easy presentation: where is your pain, what type of medication do you take and can you take, difficult to navigate.*
POP Portal not user friendly via mobile site	Patient reports having technical issues with the POP Portal's mobile site.	1	*This is a good resource for someone who owns a computer and uses it; not so much for someone who uses their phone.*
Acceptability			
Resources were helpful for patient's concerns	Patients report that the POP Portal provided resources which effectively met their concerns. These resources helped patients validate their thoughts, improve sleep hygiene, and provided hope.	9	*Sleep resources from sleepwell.ca were useful for me; I don't have a nighttab and need to get off my phone before going to sleep.*
			*Negative emotion causes more pain for me. The Portal helped with my mood and validated my thoughts and feelings about pain. I want to use even more resources on it.*
			*There is so much useful information on there. I struggled with terrible pain last summer for about 6 months and would have loved having access to the Portal and its resources. From the videos, articles etc. there is lots of info in there.*
			*Information on the use of opioids and different ways to alleviate pain, instead of using narcotics, was useful.*
			*I used Empowered Management and it's very validating of my understanding of my pain with information for self-actualization and realization.*
			*I would recommend this to people who have chronic pain. You get distracted from your pain while using the Portal's resources and it gives you hope.*
			*There are a lot of tips on the Portal. I don't know how long people have had pain for and what causes it but think everybody should read the Portal's resources and learn about chronic pain and resources to tackle it.*
			*I found the Portal's resources that describe the different types of chronic pain and how they apply to my life to be the most useful.*
			*if I can engage with the Portal when I get terrible pain, I would read articles the most.*
Improves understanding of pain	Patients report that many POP Portal resources offer pain education that further grew their understanding of pain and pain management.	10	*The pain neuroscience content was good for understanding the brain and pain's relationship; also a cartoon that shows wolf/raccoon has connection between the brain and pain; video of professor dying from brown snake bite and then had no pain was interesting.*
			*Pain is a personal experience, I learned this from resources like tame the beast.*
			*"Pain is all in the mind” is untrue; I learned about our danger receptors. I use Taiichi to calm my brain down and do yoga.*
			*Going through pain concepts/definitions and resources are good and I am liking it.*
			*Empowered Management modules and Portal resources that are most useful to me are about pain and managing pain. It helps me understand these and modules that ask me to do some type of activity*
			*The Portal's content is fairly educational. I enjoyed several of the videos on there; it provides good basic information and sets out things well. Chronic vs. acute pain is explained well. LPB+ has the same video with another expert area (got tired from repetition of the videos).*
			*If you're waiting for a miracle to happen with your pain management person, that's not good; this Portal can help you; it also provides a lot of education in advance of your visit with healthcare providers.*
			*Talking about the cycle of pain gave me a better understanding of pain than high-level research studies/topics.*
			*If you're new to pain management with trying pain pills or seeing healthcare providers, this is a good resource for you.*
			*But the Portal is a good first resource for people who haven’t tried much interventions or don’t know much about chronic pain and management.*
Patient enjoyed suggested resources after self-assessment	Patient enjoyed having the ability to see resources tailored to their self-assessment results.	3	*Self-assessment scores can be linked suggested resources for mental health and pain management.*
			*I was impressed with the account's dashboard and suggestions for improving chronic pain symptoms; the way the questions were set up on self-assessments are good.*
			*Great that there are interventions being suggested to a user by the Portal based on their self-assessment scores.*
Did not offer new information	Patient reports no new information was presented on the POP Portal.	2	*I knew most of the information on the Portal and most of it doesn't apply to my condition.*
			*When I speak with my doctor, they give me all the info that is on the website and I live with a doctor and have extensive knowledge.*
Was not suitable for patient	Due to current condition, patient does not believe the POP Portal is helpful for their current needs (e.g. medicine)	6	*I am looking for medical help mainly. The PoP Portal is good but I don't think it can help me at this time.*
			*I am working on seeing a surgeon for my pain. I don’t think the Portal would be useful at this time. I may revisit it in the future.*
			*I think pain education is a good thing, maybe some will get what they need out of it on the Portal. My condition is a bit unique and so far, all conventional pain management techniques (minus medication) have not worked and it's a medical case.*
			*Reading doesn't help me much; I want to talk to people*
			*I did the self-assessments but a lot of them don't apply to me at this time, e.g. mood questions are not related to my condition.*
			*I did the self-assessments on the Portal but feel that the questions don’t apply to me for the most part. Nerve pain intensity stays the same and I don't want to follow up with the self-assessments as I feel that they are not capturing my symptoms well and also my pain intensity doesn't change; I only have pain in the morning; throughout the day, I am okay and pain has no impact on my daily activities. I still have limited sleep quality.*
Patient Value of the POP Portal			
POP Portal can be helpful at all treatment stages	The POP Portal is helpful for various stages of pain management, including previsit and during treatment.	2	*It's an excellent idea to use the Portal for PLWP waiting for their first appointment at a pain clinic because of the type of resources on it; I think even during their appointment with pain clinic, they could also maybe be introduced to the Portal's resources.*
			*When I had vascular disease in my leg and had surgery, there were no resources at all for me like the Portal; I hope they can integrate this model in their division.*
Self-assessments were helpful to track progress	Patients used the POP Portal to track their progress and pain symptoms over time.	2	*I have been journaling all the self-assessment questions. I find it soothing and my mind gets a break.*
			*I've started journaling my pain journey using the Portal's self-assessments.*
Offers pain management while waiting for first visit	The POP Portal offers a form of chronic pain management while awaiting a first visit to a tertiary pain clinic.	2	*Personally, I believe the Portal helps because I am not just sitting around writing while waiting for first appointment at the pain clinic; at least I can try and manage pain while I wait.*
			*Mental health is involved when you're waiting for care and when you are in pain, people may give up, then end up at the hospital and the psychiatric appt and suicide rates will go up. Many people are on disability and there's no one to care for them while they wait for a first appointment at the pain clinic, but have the Portal to rely on.*

#### Accessibility of the portal

Users indicated that the POP Portal offers a variety of resources that were easily accessible. Many PLWP expressed their excitement and enthusiasm to have readily available resources at their fingertips: “*There's no other website that offers so many resources all in one place; I liked this about this platform.”*

Several users suggested additional resources that are not presently available to be included on the Portal, including: 1) medication education resources; 2) time management courses; and 3) 1:1 consultation with a pain specialist: “*A suggestion for improvement would be virtual access to a pain specialist, but I realize there are complications in that being a reality. I think the portal is useful, in terms of providing information, however most people are going to want some guidance from a human being.”*

#### Usability of the portal

Patients offered suggestions to improve user experience, including additional features: “*If your Portal offered something like this [a diagnosis-specific filter] for users when they start using it, it would be useful: I have a sore back and these are the causes and the Portal directs you to these areas.”*

One patient mentioned that some aspects of the Portal did not meet their standards of usability, including difficulty with visuals presented on the Portal. The patient noted difficulty reading text due to the size of the font.

A few users highlighted the limitation of the Portal technology requirements. One user said, “*This is a good resource for someone who owns a computer and uses it; not so much for someone who uses their phone.”*

#### Acceptability of the portal

Some patients indicated that the POP Portal was helpful. “*Going through pain concepts/definitions and resources are good and I am liking it.”* Some highlighted specific resources (e.g. pain neuroscience resources, sleepwell.ca, Empowered Management) which contributed to their positive experiences: “*Negative emotion causes more pain for me. The Portal helped with my mood and validated my thoughts and feelings about pain. I want to use even more resources on it.”*

Others commented on the usefulness of the features available on the Portal. “*I was impressed with the account's dashboard and suggestions for improving chronic pain symptoms; the way the questions were set up on self-assessments are good.*”

Some patients believed that the POP Portal was not suitable for their current needs. Some users reported time constraints as a barrier, while other users reported seeking medical interventions and 1:1 treatments that were not included on the POP Portal*. “I am looking for medical help mainly. The POP Portal is good but I don't think it can help me at this time.”*

#### Patient value of the POP portal

Some patients reported benefit to accessing the POP Portal while waiting for care at the clinic, and all recommended we continue to offer the POP Portal for those who are waiting: “*If you're waiting for a miracle to happen with your pain management person, that's not good; this Portal can help you; it also provides a lot of education in advance of your visit with healthcare providers.”*

Others endorsed the POP Portal and encouraged others to avail of it, including PLWP, friends/family, and healthcare professionals: “*I would recommend this to people who have chronic pain. You get distracted from your pain while using the Portal's resources and it gives you hope.”*

Some highlighted the uniqueness of the Portal, indicating the centrality of resources: “*There's no other website that offers so many resources all in one place; I liked this about this platform.”*

## Discussion

The present study explored the initial implementation of the POP Portal in the referral pathway at a tertiary pain clinic and evaluated the experiences of PLWP using the Portal while waiting for care, acting as the first evaluation of acceptability for the POP Portal. Findings suggest that some patients waiting for care at a tertiary pain clinic who were introduced to the POP Portal found it: 1) enhanced access to a variety of online resources in one a centralized location; 2) helpful in their journey to self-management; and 3) potentially beneficial for anyone who is waiting for pain care at a tertiary car pain clinic. Although the POP Portal was found to be helpful for most participants in this study, individuals who looked for primarily medical interventions and high-intensity services (i.e. 1:1 support from a physician or pain specialist) did not feel as though their needs were met when using the POP Portal. Suggestions were made to improve the POP Portal to be inclusive of differing pain diagnoses and increase user engagement with resource materials by addressing shortcomings in Portal usability.

One of the major impetuses behind the POP Portal is to address the unmet needs of patients referred to tertiary CP care clinics through a person-centric approach that provides a stepped-care continuum of resources for self-management of pain, mental health, and substance use health. From this initial implementation project, the POP Portal was reported by PLWP to satisfy their pain management needs in an accessible way while they waited for an appointment at TOH Pain Clinic. Additionally, the POP Portal is accessible internationally to any person living with pain. At end of March 2023, the POP Portal reached a total of 1852 users. More recently, as of June 2024, the POP Portal reached a total of 233,243 users across the world, of which 227,200 were Canadian users. The POP Portal increases access for clients by curating evidence-informed CP self-management resources in a single website. Platforms such as the POP Portal have shown acceptability and usability among patients. A meta-analysis and systematic review that included 67,884 patients across 65 studies indicated that the use of web-based programs (e.g. psychoeducation, support groups, CBT) reduces barriers of participation in care, allowing higher patient retention and satisfaction, and greater program usability when compared to in-person equivalents.^21^ Another study reported that patients attending a physical self-regulation program at an orofacial pain clinic in-person were less likely to complete all three sessions than those who completed via telehealth.^22^ This may suggest that digital resources, such as those seen on the POP Portal, offer convenient access to care by removing influential barriers. One difference that separates the POP Portal from other web-based programs is the diversity of resources available to the user. The POP Portal offers not only diverse resource types (i.e. articles, videos, webinars, workshops, peer support, etc.) but it also reaches beyond CP to provide resources touching on topics that are often a top-of-mind concern for PLWP, including sleep hygiene, medication, diagnosis, financial supports, indigenous supports, and mental health. Centralization of resources acts to reduce barriers PLWP may experience when seeking pain self-management, as all of the resources they may be interested in at that moment in time are readily available, free of charge, in one convenient location with no need to search website to website which can be time-consuming and frustrating for the user.

Further, some patients have indicated that the use of resources on the POP Portal, including sleepwell.ca and the self-directed course Empowered Management, have been helpful in reducing symptoms associated with pain (e.g. mood, self-efficacy). Internet-based pain management interventions, including iCBT and iACT, have been shown in many studies to improve pain intensity, pain catastrophizing, and symptoms of anxiety and depression.^9,23–25^ A randomized control trial comparing patient outcomes of iCBT to a waitlist control reported improvements to disability, depression, anxiety, and pain intensity greater than 30% in the treatment group and was sustained at three-month follow-up.^9^ It may be beneficial within a broader context to introduce self-management resources, such as self-directed courses, as a complimentary step within an individual's CP management and preparing the individual for treatment in a tertiary care clinic. As many waitlisted patients may experience a deterioration in their condition as they wait for a first appointment,^26^ access to such resources may be beneficial for maintaining their condition until they receive treatment from a healthcare professional.

People living with pain have endorsed the POP Portal as a platform which provides a variety of resources for pain management; however, some have indicated that the POP Portal does not provide resources addressing the needs associated with living with specific chronically painful conditions. Research conducted on digital pain management websites have indicated that the availability of a variety of resources is important for acceptability and engagement, specifically highlighting the need for diagnosis-specific resources.^27^ While many CP self-management strategies are appropriate across several chronically painful conditions, CP is complex and some conditions leading to CP may benefit from more tailored approaches.^16^ As such, the inclusion of diagnosis-specific resources on a digital platform such as the POP Portal may help to improve acceptability and engagement across a larger population and should be considered as the POP Portal continues to evolve.

### Future directions

Our goal is to leverage the experiences of PWLP using the Portal during initial implementation to improve the POP Portal in an iterative manner to better suit the needs of PLWP. Results of the present study will be used to inform changes to the POP Portal, including additional resources and improved usability. We will also continue to work on the implementation of the POP Portal and study a range of patient, implementation, and health system outcomes. This will include the next step of this project, a hybrid implementation-effectiveness type III pilot study to evaluate the feasibility of conducting a future definitive trial. The trial will provide important information pertaining to the acceptability and usability of the POP Portal using validated measures, as well as exploration of the Portal's impact on patient outcomes after three months of use. Further, future research will explore the need for diagnosis-specific resources to inform future development of the POP Portal. We aim to integrate participant feedback into the POP Portal in an iterative process so that the Portal may continue to grow to fit the needs and preferences of PLWP. Additional research will explore the integration of the POP Portal into clinical practice, highlighting the barriers and facilitators of implementation, healthcare provider perceptions of the POP Portal, and factors which may impact long-term sustainability in clinical practice. We will also conduct research to determine the characteristics of users within each resource and further evaluate the acceptability and usability of the resources across age, diagnosis, sex, and gender. Finally, we will explore the implementation of the POP Portal within primary care settings, evaluating the benefits, barriers, and facilitators of implementation, as well as the development of a decision-support intervention to support sustainable integration of the POP Portal within primary care pathways.

### Study limitations

The use of qualitative methods built on theoretical frameworks and with sufficient sample size for information power (*N *= 40) to describe perceptions of PLWP about the POP Portal; however, it would be beneficial to expand this quality improvement study to a multisite project to account for potential selection bias and allow for greater transferability among PLWP. Further, the present study provided first insights of acceptability and usability of the POP Portal when interviewing participants; however, no validated measures were used. As such, information gathered from this study should be interpreted with caution until additional research is conducted in the next step of this project, where validated measures will be used to evaluate the acceptability, usability, and feasibility of the POP Portal in a larger sample. Additionally, we are uncertain what resources the participants may have used while participating in the study. Each participant would have had to make an account and collect demographic data on the Portal, which was not enforced for this study. As such, it is difficult to determine which resources had the greatest reach among participants and the associated characteristics. This is a step which may be taken in future research to evaluate frequency and patterns of Portal use. Further, the present study does not have a measure of engagement, such as the length of time participants had spent using the POP Portal. This is an important measure to consider when evaluating acceptability and usability of a digital intervention and will be included in future research. Finally, the present study used field notes to document participant thoughts toward the POP Portal during interviews. As these are not direct quotations, we suggest caution in the interpretation of these quotations as they represent the interpreted sentiment of the participant.

## Conclusion

Overall, the present study conducted semistructured interviews with PLWP who have used the POP Portal while waiting for care at a tertiary care pain clinic. Present data illustrate most participants found the POP Portal helpful for their concerns and gaining an understanding of CP. The POP Portal also met their standards of accessibility, providing easy access to pain self-management when it is needed most, although improvements may be made to provide additional resources and meet usability needs.

## Appendix A: Interview Guide

### Participant's use of the POP portal in the past four weeks

1. Over the past month, did you get to go on the Portal and use it?

### Participant's experience using the POP portal

2. Tell me about a time you turned to the Portal (when was it, what did you use, how was it)
3. As a result of using the Portal, did you learn anything new?
4. Did you change anything in your pain management?
5. Did you create an account on the Portal?
6. Did you complete any self-assessments? If yes, how many?
7. Which resources did you use?
8. Which resources did you find to be most beneficial for you?

### Future use of the POP portal

9. Do you think that we should continue to use the Portal as a resource for people waiting for a first appointment at a pain clinic?
10. Do you intend to use the Portal in the future?
